# Can you feel meaning? Spirituality involves stronger sensorimotor associations with abstract concepts

**DOI:** 10.1098/rsos.260997

**Published:** 2026-10-28

**Authors:** Natalia Zarzeczna, Travis Proulx, Brian Meier, Eva Gutierrez-Sigut

**Affiliations:** ^1^ Department of Psychology, University of Essex, Colchester, UK; ^2^ Cardiff University, Cardiff, UK; ^3^ Gettysburg College, Gettysburg, PA, USA; ^4^ University of Essex, Colchester, UK

**Keywords:** abstract concepts, meaning, spirituality, sensorimotor grounding, grounded cognition

## Abstract

Understanding abstract phenomena is a fundamental human need. Emerging evidence increasingly points to the multidimensionality of abstract knowledge representations, with theories emphasizing the importance of both embodied and linguistic influences. In the present paper, we argue that the strength with which embodiment is associated with abstract concepts is moderated by cultural beliefs about ontological meaning. Specifically, we predicted that self-identified spirituality—a belief system guided by an experiential approach to understanding reality—contributes to greater sensorimotor associations with abstract concepts. As hypothesized, across four studies (*n* = 729; in the USA and the UK), Western spiritual beliefs involved stronger sensorimotor associations for highly abstract compared to concrete concepts. Importantly, religious beliefs that conceptually overlap with spirituality did not contribute to these associations. Altogether, individual differences in belief systems guide people's conscious attendance to sensorimotor features when thinking about abstract concepts, with spiritual individuals likely feeling the meaning of words through linking abstract thought with bodily experiences.

## 1. Introduction

Understanding reality is a fundamental human need. Beyond epistemic concerns about the physical world, people are strongly motivated to understand complex abstract phenomena, such as the nature of existence. Yet, abstract conceptual understanding remains a challenge for theories of cognition. The influential embodiment perspective, which emphasizes the importance of perception, action and emotion systems in determining the meaning of words, cannot fully account for the complexity of abstract concepts [1]. Indeed, brain networks associated with sensorimotor experiences are typically active when thinking about concrete concepts, but abstract concepts primarily involve linguistic activation (see [2] for an overview). On the other hand, linguistic distributional semantic theories, which consider meaning to be derived from word associations, suffer from the symbol grounding problem. That is, symbols need to be understood in relation to their real-world referents, rather than other abstract symbols (see [3] for an overview). More recent hybrid theories, however, account for the role of both linguistic and embodied influences [4]. Importantly, current evidence increasingly points to the multidimensionality and flexibility of abstract concepts by acknowledging the contribution of socio-cultural context or individual differences in sensory experiences [2,5–7]. Building on this literature, we argue it is possible that while sensorimotor grounding contributes to the representation of abstract concepts, its strength depends on individual differences in cultural belief systems. In the present paper, we begin addressing this issue by examining whether spiritual beliefs about ontological meaning contribute to increased conscious attendance to sensorimotor features in abstract concepts.

Spirituality has recently grown in prevalence in Western countries [8,9]. Although Western spiritual beliefs are heterogenous, a common aspect that binds them together is an experiential approach to ontological meaning, that is, the belief that truth is subjective, personal and can be found by looking inwards [10]. This experiential way of knowing is linked to the conviction that one can feel what is true [11]. As spirituality emphasizes the importance of subjective experience in understanding reality, we examine whether this approach contributes to sensorimotor associations with abstract knowledge. As an additional aim, we explore whether sensorimotor associations have implications for objective approaches to knowledge involving science.

## 2. Flexibility of abstract concepts

Understanding the meaning of abstract concepts is essential for navigating, predicting and controlling the physical environment, therefore imposing coherent meaning on the social world. Yet, the nature of the mental representation of abstract concepts has been challenging to study and highly debated. Influential theorists of embodied cognition perspectives postulate that conceptual representations are grounded in perception, action and introspection, rejecting earlier theories of cognition that conceptualized knowledge as amodal [12]. In other words, knowledge representation is seen as tied to bodily experiences, such as those with sensory modalities (e.g. vision) or motor effectors (e.g. action with legs/arms), and involves a simulation or reenactment of these previously acquired experiences. While ample evidence supports this view for concrete concepts, which have clear perceptual qualities (e.g. bottle), embodied cognition cannot fully account for the representation of abstract concepts, which lack clear physical referents in the environment (e.g. meaning) [1]. Indeed, compared to concrete concepts, abstract concepts are more complex, as they are largely detached from perception and have low iconicity (i.e. little correspondence between the word's form and its meaning), dimensionality (i.e. sharing few features) and imageability and are difficult to understand, with people disagreeing more about their meaning [5,6,13]. As such, abstract concepts are less grounded in sensorimotor experiences compared to concrete ones, exposing limitations of the embodiment perspective [1]. Indeed, recent theories strongly emphasize the importance of linguistic input and social metacognition (i.e. a reflection on needing other people to explain the meaning of words), rather than the exclusive reliance on direct sensorimotor experiences with referents in the physical environment [14].

Although embodied cognition cannot fully explain the mental representation of abstract concepts, this does not preclude the possibility that various abstract concepts are still linked to sensorimotor experiences under certain circumstances. Specifically, the strength of the embodiment-abstract thought link depends on the nature of the represented concept, type of perceptual or motor experience and contextual and physical features of the environment. As an example, both types of concept categories, concrete and abstract, are differently tied to sensorimotor experiences. Although visual modality often dominates both categories [13], concrete concepts are particularly tied to haptic experience and hand–arm movement, while abstract concepts involve auditory and interoceptive experiences [13]. Further, highly abstract philosophical–spiritual concepts (e.g. mystery, religion, virtue and logic) involve particularly weak sensorimotor associations, but abstract concepts associated with self and sociality are strongly grounded in inner bodily sensations (i.e. interoception) [7]. Overall, while abstract concepts have less prominent sensorimotor representations, there are contextual factors that moderate how strongly abstract concepts are tied to sensorimotor dimensions.

Finally, the representation of abstract concepts depends on contextual online cues rather than solely fixed long-term memory links between phenomena and sensorimotor experiences [15]. Evidence suggests that experiences with concrete concepts, such as physical space, can be used as a flexible toolbox in the representation of a range of abstract concepts, consistent with conceptual metaphor theory [5,15,16]. As such, highly abstract reasoning about coherence – the experience that a set of concepts makes sense together as a whole—involves flexible and online associations with upper vertical positions and spatial proximity. These associations depend on the availability of specific spatial cues in the environment [15,17]. Likewise, other abstract concepts related to valence, morality, power, magnitudes (e.g. dominance), social stereotypes or religious entities are associated with the horizontal or vertical spatial dimensions [18–25]. Again, this evidence suggests that even though abstract concepts lack physical referents, concrete concepts associated with physical dimensions may be used to facilitate thinking about abstract concepts, linking abstract representations to concrete sensorimotor experiences.

Altogether, the extent to which sensorimotor experiences are involved in the representation of abstract concepts strongly depends on the context, types of concepts and perceptual or action dimensions. However, beyond these factors, we argue that individual differences in higher-order cognition moderate the strength of sensorimotor associations in abstract concepts. That is, our proposal is that the extent to which abstract thoughts involve sensorimotor features also varies depending on one's attitudes towards ontological meaning. Recent empirical insights suggest that conceptual representation is more nuanced than previous theories of cognition assumed, with individual differences and diversity of experiences being important factors in such representation [5]. Although evidence in this area is still scarce, studies demonstrate that semantic networks change with age owing to cognitive learning and experiences, while socio-cultural norms influence the way people define concepts such as gender [26–28]. In this light, we propose that spiritual belief, in its focus on deriving ontological meaning from inner and bodily experiences, is particularly relevant to conscious attending to sensorimotor features.

## 3. Spiritual quest for meaning

Within historical accounts, spirituality is defined as a quest for absolute meaning, or in other words, *true* knowledge about reality and existence [10]. In other words, discovering spiritual truth refers to gaining awareness of unknowable or hidden elements that exist outside of human senses (i.e. Kant's *noumena*). Despite the pluralism of esoteric beliefs, spirituality largely posits that such spiritual meaning is hidden in the divine properties of one's soul which are not readily accessible to the individual [29]. The main human quest is therefore to discover these properties through spiritual practices involving a focus on subjective experiences which grant access to ontological meaning. Overall, spiritual epistemology, termed *gnosis*, relies on the idea that truth can be found within oneself and hence stands in contradiction to external forms of knowledge, including both dogmatic religious faith and positivist scientific reason [29,30].

These insights about spiritual beliefs are well supported by recent evidence illustrating that commitment to spiritual beliefs contributes to existential meaning and represents a distinct approach to ontological knowledge beyond religiosity [31]. First, those who identify as spiritual but not religious report greater meaning in life (in terms of coherence, significance and purpose) than secular individuals, a difference explained by commitment to mystical beliefs that are associated with a sense of order and connection in the universe [32]. Second, spirituality is a distinct way of knowing that prioritizes inner as opposed to external epistemology. Indeed, self-identified spirituality is consistently linked with a greater reliance on intuitive approaches to knowledge, including trusting gut feelings, beliefs that one can feel what is true and one's inner knowing is superior to other people's knowledge [11,31,33,34]. That is, although spirituality can be experienced in relation to religious beliefs, such as personal experiences with a deity, Western spirituality often functions independently and outside of religious traditions [29].

As such, ample evidence demonstrates that spirituality has unique consequences for societal challenges. Even when controlling for religiosity, spiritual beliefs predict low trust in objective scientific method as a way of discovering ontological meaning, and such low trust explains stronger science scepticism, particularly towards vaccination (e.g. distrust in the safety and effectiveness of vaccines [11,34–39]).

Altogether, given that spiritual individuals consider subjective inner experiences as cues to ontological meaning, we propose that they may be predisposed to representing abstract objects and phenomena in the context of their bodily experiences and therefore consciously associate these concepts with sensorimotor features. We therefore conceptualize sensorimotor associations as cognitive characteristics of an inner knowing experience. As epistemological conflicts between scientific and spiritual approaches to knowledge contribute to stronger vaccination scepticism [38], we additionally hypothesize that sensorimotor associations would exacerbate vaccination rejection in spiritual individuals. As such, we test whether stronger sensorimotor associations with abstract concepts predict greater science rejection among spiritual individuals.

## 4. The present research

Integrating insights from grounded cognition with spiritual approaches to ontological meaning, we hypothesized that stronger spirituality would involve greater sensorimotor links with abstract concepts, and this would be especially pronounced for highly abstract concepts owing to their higher representational flexibility [14,29], and when controlling for other relevant variables, including religiosity, political beliefs and demographics. We assessed this using general concrete and abstract concepts (ball/democracy) and those relevant to spirituality (crystal/transcendence). Importantly, spiritual and philosophical concepts, which are highly abstract, were previously found to be weakly associated with sensorimotor experiences [7]. However, as highly abstract concepts are especially variable across contexts, we suggest that individual differences in belief systems would strongly guide conscious sensorimotor associations for such concepts [5]. As an additional aim, we also explored whether stronger sensorimotor grounding contributes to increased vaccination scepticism among spiritual individuals [38]. It is possible that more pronounced links between sensorimotor experiences and concepts function as a facilitatory mechanism for intuitive ways of thinking that increase science rejection.

We tested the hypotheses in four online studies. Across all studies, we measured participants’ sensorimotor associations with a series of concepts. Participants rated the extent to which they experienced each concept with sensory/perceptual modalities (auditory, gustatory, haptic, olfactory, visual and including interoceptive experience) as well as motor effectors (foot, hand, head, mouth and torso) on a scale. In all studies, we selected concepts that were relevant to spiritual beliefs that were both concrete (e.g. body and crystal) and abstract (e.g. transcendence and purpose). In Study 2, we also used general concepts that were abstract (idea and hope) and concrete (ball and tree). Across all studies, we measured participants’ self-reported beliefs, including self-identified spirituality (and additionally post-Christian spirituality in Study 1), religiosity, political conservatism and demographics. In Studies 2–4, we assessed whether sensorimotor grounding contributes to greater vaccination scepticism among strongly spiritual individuals.

## 5. Study 1: Sensorimotor associations with spiritual concepts

### 5.1. Methods

#### 5.1.1. Transparency and openness

We follow JARS in reporting all studies in the present manuscript [40]. We report how we determined sample sizes, we list all exclusions, and we are transparent about all measures. All study materials, datasets and analysis code are available on the Open Science Framework at https://osf.io/v5mc4. Additionally, the hypotheses, materials and analysis code were pre-registered in Study 3 at https://osf.io/ug8jh.

#### 5.1.2. Participants

We recruited 149 participants in the US through the online recruitment platform Prolific. The sample size was estimated based on a power analysis for a multiple regression analysis with two tested predictors (participants’ spirituality and concept concreteness), with a total of seven predictors, including the interaction term between spirituality and concreteness, for a small effect size *f* = 0.10, an alpha = 0.05 and 80% power. We note that we conducted a power analysis for a multiple regression analysis rather than a linear-mixed model, as we did not have prior data to conduct a simulation power analysis. The analysis estimated 100 participants, but we aimed to recruit at least 150 to compensate for potential data loss. We excluded participants who failed at least one attention check, leaving the total sample size at 141 participants. Demographic information about participants is presented in table 1. Participants received £2.20 as compensation for a 15–20 min survey. The study received the University of Essex ethics approval: ETH2324-1635.

**Table 1.** Samples' characteristics across all studies.

variables	Study 1	Study 2	Study 3	Study 4
*n*	141	96	139	351
nationality	US general population (prolific sample)	UK student sample	UK student sample	UK general population (prolific sample)
language	English native speakers: 97%	English native speakers: 65%	English native speakers: 84%	English native speakers: 100%
age (years)	*M =* 37.8 s.d. *=* 11.7 range: 19–74	*M =* 21.2 s.d. *=* 4.42 range: 18–56	*M =* 19.5 s.d. *=* 1.40 range: 18–28	*M =* 44.80 s.d. *=* 1.28 range: 20–78
gender	women: 51% men: 48% non-binary: 0.5% prefer not to say: 0.5%	women: 83% men: 16% gender fluid: 1%	women: 85% men: 15%	women: 56% men: 43% prefer not to say: 0.5% missing: 0.5%
political orientation (1 = *liberal*; 10 = *conservative*)	*M =* 5.14 s.d. *=* 3.01	*M =* 4.23 s.d. *=* 1.66	*M =* 3.43 s.d. *=* 1.64	*M =* 4.50 s.d. *=* 1.90
religiosity	*M =* 3.74 s.d. = 2.09	*M =* 3.10 s.d. *=* 1.97	*M =* 2.41 s.d. *=* 1.80	*M =* 2.05 s.d. *=* 1.63
spirituality	*M =* 4.37 s.d. = 1.89	*M =* 3.35 s.d. *=* 1.74	*M =* 3.12 s.d. *=* 1.55	*M =* 2.98 s.d. *=* 1.81

#### 5.1.3. Material

##### Concepts

We aimed at selecting 30 concepts that were relevant to spiritual beliefs, with an equal number of concrete and abstract. To achieve this, we used Villani *et al.*'s [7] abstract and spiritual concepts database, with content focused on spirituality-related beliefs also derived from previous research on esotericism in history, philosophy and psychology [7,30,38,41]. We selected spiritual-concrete and spiritual-abstract concepts based on concreteness ratings obtained from the concreteness norm database of 40k English words [42]. The concepts that were relevant to spirituality and which had a concreteness norm rating of more than 2.5 (on a five-point scale) were considered concrete^1^ (*M* = 3.41, s.d. *=* 0.73, range: 2.76–4.80) (*n* = 14; body, connection, cosmos, crystal, earth, energy, force, growth, healing, inner, meditation, nature, self and universe), while those below 2.5 as abstract (*M* = 1.76, s.d. = 0.33, range: 1.07–2.48) (*n* = 16; astrology, awe, holism, experience, introspection, intuition, meaning, purpose, sacredness, significance, soul, spirituality, transcendence, unity, universalism and wonder).^2^ Frequency of the concrete concepts was slightly higher (*M* = 9844, s.d. = 9814, range: 370–30 204) than the abstract ones (*M* = 6598, s.d. = 9082, range: 7–28 567) based on the SUBTLEX-UK frequency database for English words [43].

##### Concept concreteness

We used continuous concreteness ratings for each concept (derived from [42]) as a moderator (dichotomizing the ratings into concrete and abstract categories did not affect the results).

##### Sensorimotor associations measure

The sensorimotor measure was directly derived from Lynott *et al.* [44] and comprised questions tapping into experiencing concepts with six perceptual modalities and five motor effectors. Specifically, on a scale from 1 (*not at all*) to 6 (*greatly*), participants rated the extent to which they experienced each concept with each perceptual modality by feeling through touch, hearing, seeing, smelling, tasting and sensations inside their body, and by performing an action with effectors: hand/arm, foot/leg, torso, mouth/throat and head excluding mouth. Each effector was illustrated with a picture of a specific body part it referred to. All concepts were grouped into two blocks: perceptual modalities and motor effectors. The blocks’ presentation order was counterbalanced between participants. The presentation order of items within each block was randomized. For descriptive purposes, for the polysemic concepts (*n* = 3) with multiple meanings (i.e. energy, connection and wonder), we additionally asked participants what precisely they had in mind while thinking about experiencing these concepts to assess whether participants considered them as concrete or abstract. The majority of participants (over 90%) considered all concepts more abstract than concrete (e.g. by reporting thinking of connection in terms of relationships rather than physical connection between objects). None of the participants were excluded based on these responses. We also included two attention checks in each block, where we asked participants to indicate 1 for all modalities and effectors (e.g. This is a test item. Please select 1 for all senses to show that you are paying attention).

##### Spirituality

As spiritual beliefs are highly diverse and individualized, we aimed to capture a wide range of spiritual beliefs. For this reason, we measured self-identified spirituality by asking participants the extent to which they consider themselves spiritual and whether others consider them as spiritual (1 = *not at all*, 7 = *very much*) (*r* = 0.86) [45]. This two-item measure showed convergent and predictive validity in previous studies [31,34,38]. However, we additionally measured post-Christian spirituality [46] (‘The divine does not originate outside, but within every person’, 1 = *strongly disagree*, 5 = *strongly agree*, 0 = *don't know*; *α* = 0.88) to ensure that our findings replicate using an alternative measure. Both spirituality measures (the two-item measure and post-Christian spirituality) were correlated, *r* = 0.40. These measures were intended to measure self-identified spirituality and an inner epistemology, rather than spiritual rituals or practices.

##### Religiosity

We measured religiosity with a two-item measure similar to the measure of spirituality, where participants indicated their self-identified religiosity (‘I consider myself as a religious person’ and ‘Others consider me as a religious person’). The items were reliable (*r* = 0.89).

##### Demographics

Participants also completed measures of political ideology by indicating the extent to which they described themselves as liberal or conservative on a scale (1 = *liberal*, 10 = *conservative*), reported age, gender and whether English was their first language.^3^

### 5.2. Results

We first computed zero-order correlations between sensorimotor associations and beliefs (table 2). Second, we estimated a mixed-effects model with sensorimotor associations as the outcome, while spirituality, concept concreteness, and the spirituality and concept concreteness interaction as the main predictors. We also controlled for category (sensory or motor). We included a range of other control variables, that is religiosity, conservatism, age and gender. Random intercepts were specified to vary across participants and concepts. All predictors and the outcome were standardized. All analyses were conducted in RStudio (v.2024.04.1+748) using lme4, interactions and emmeans packages. The model structure was as follows: lmer(sensorimotor rating ∼ spirituality × concept concreteness + category + religiosity + conservatism + age + gender + (1 | participant) + (1 | concept)). The results are presented in table 3.^4^

**Table 2.** Zero-order correlations between beliefs and sensorimotor associations (Study 1).

	1	2	3
1. spirituality			
2. religiosity	0.64**		
	[0.54, 0.73]		
3. conservatism	0.21*	0.40**	
	[0.05, 0.37]	[0.25, 0.53]	
4. sensorimotor associations	0.41**	0.28**	0.03
	[0.26, 0.54]	[0.12, 0.43]	[−0.14, 0.19]

**p < 0.001, *p < 0.05.

**Table 3.** Predictors of sensorimotor associations (Study 1).

	sensorimotor associations		
predictors	*β*	95% CI	*p*
spirituality	0.22	0.11–0.32	0.001*
concept concreteness	0.05	−0.05 to 0.15	0.294
category (motor: 0, sensory: 1)	0.00	−0.01 to 0.01	0.959
religiosity	0.03	−0.08 to 0.14	0.616
conservatism	−0.04	−0.13 to 0.05	0.343
age	0.03	−0.05 to 0.11	0.434
gender (1: women)	0.07	−0.01 to 0.15	0.085
spirituality × concept concreteness	−0.03	−0.03 to −0.02	0.001*
**random effects**			
*σ*^2^	0.64		
*τ*_00 ps_	0.23		
*τ*_00 item_	0.07		
ICC	0.32		
*n*_ps_	141		
*n*_item_	30		
observations	45 040		
marginal *R*^2^/conditional *R*^2^	0.060/0.365		

*p < 0.001.

#### 5.2.1. Confirmatory analysis

We found that spirituality significantly predicted stronger sensorimotor associations, after controlling for other relevant beliefs, including religiosity and political orientation (figure 1). Concept concreteness further moderated this effect, with post hoc simple slopes and trends analyses indicating that while spirituality significantly predicted sensorimotor associations, this effect was the strongest for highly abstract (−1s.d.) concepts (*β* = 0.24, 95% CI [0.14, 0.35], s.e. = 0.05, *t* = 4.49, *p* < 0.001), compared to moderate (*β* = 0.22, 95% CI [0.11, 0.32], s.e. = 0.05, *t* = 4.01, *p* < 0.001), estimate = 0.03, s.e. *=* 0.004, *p* < 0.001, and highly concrete (+1s.d.) concepts (*β* = 0.19, 95% CI [0.08, 0.30], s.e. = 0.05, *t* = 3.51, *p* < 0.001), estimate = 0.05, s.e. *=* 0.008, *p* < 0.001. The difference in slopes was also significant between moderate and highly concrete concepts, estimate = 0.03, s.e. = 0.004, *p* < 0.001.

**Figure 1.**
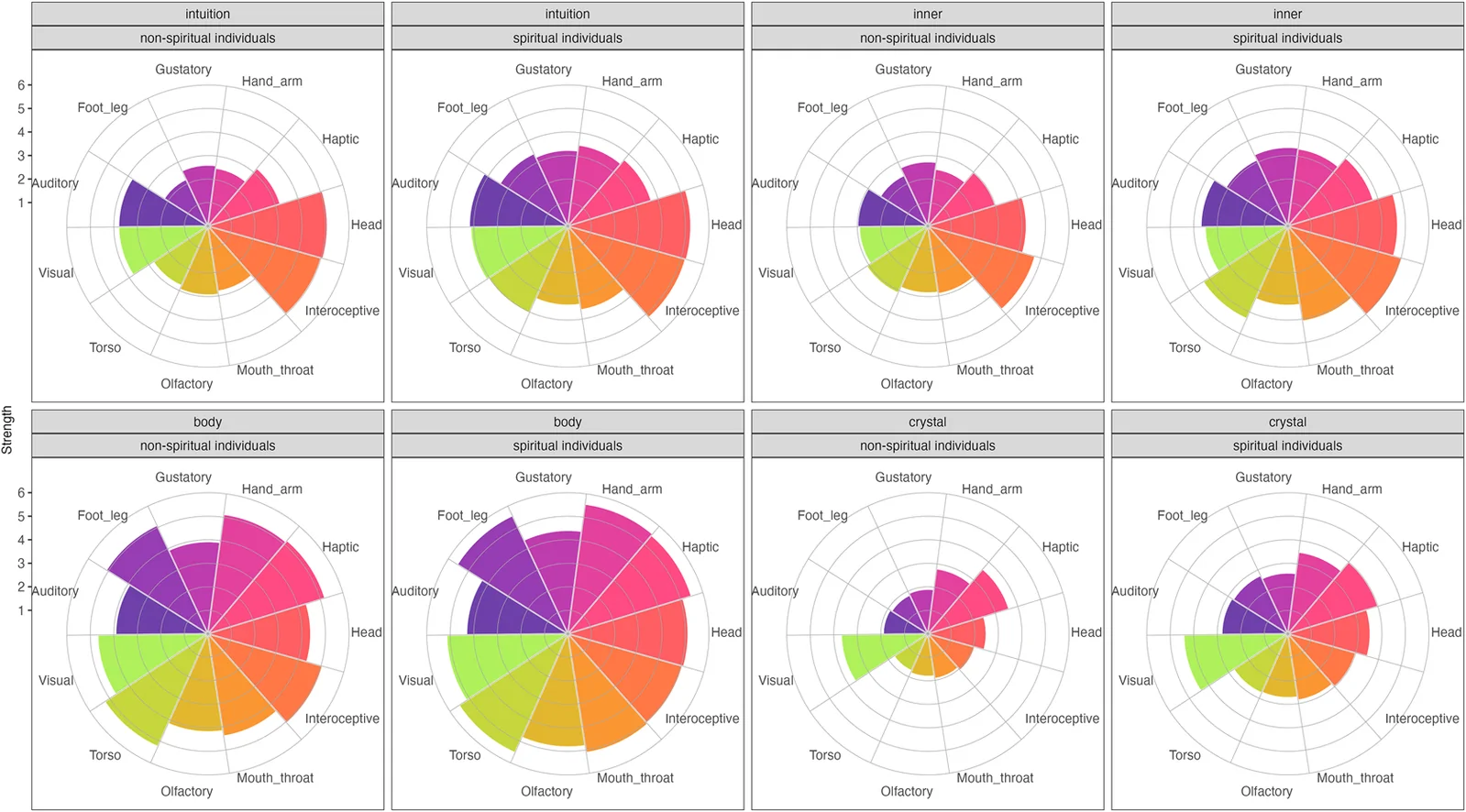
Sensorimotor strength for spiritual abstract (intuition and inner) and spiritual concrete (body and crystal) concepts for spiritual and non-spiritual individuals (median split) in Study 1.

#### 5.2.2. Exploratory analysis

##### Religiosity

First, as spirituality conceptually overlaps with religiosity, we conducted additional robustness checks. To achieve this, we estimated another model, but this time, we tested whether religiosity interacted with concept concreteness in predicting sensorimotor strength (see supplemental material for details). Across all studies, the slopes for religiosity did not significantly predict sensorimotor scores.

##### Frequency and modalities

Second, we tested the robustness of our findings by introducing word frequency as a covariate into the model: lmer(sensorimotor rating ∼ spirituality × concept concreteness + category + frequency + religiosity + conservatism + age + gender + (1 | participant) + (1 | concept). This did not change the obtained results (the effect of spirituality remained exactly the same). As sensory and motor dimensions represent distinct constructs, we also analysed these dimensions separately while controlling for individual modalities and effectors, respectively. The results were in line with the confirmatory findings indicating that greater spirituality predicted stronger sensory and motor associations separately. These findings were true across all studies (see supplemental material for full statistics).

### 5.3. Discussion

Overall, the results supported our hypothesis indicating that spirituality contributes to greater sensorimotor associations with concepts related to spiritual beliefs. These associations were the strongest for highly abstract concepts. These findings provide evidence that individual differences in approaches to meaning are reflected in low-level but conscious sensorimotor associations. In Study 2, we aimed to replicate these findings in a UK sample of participants and with a wider range of concepts to assess generalisability. In addition, we explored whether such low-level sensorimotor associations contribute to greater vaccine scepticism among strongly spiritual individuals.

## 6. Study 2: Sensorimotor associations with spiritual and general concepts

### 6.1. Methods

#### 6.1.1. Participants

We recruited 107 students using a participant pool at the University of Essex, based on the power analysis conducted for Study 1. Again, we excluded participants who failed at least one attention check, leaving the total sample size at 96 participants (see table 1 for demographic information). Participants received course credits as compensation for a 60-min study. The study received the University of Essex ethics approval: ETH2425-0120.

#### 6.1.2. Material

##### Concepts

We selected the same spiritual-concrete and spiritual-abstract concepts as in Study 1 (*n* = 30). We also selected general concepts that were highly abstract (*M* = 1.61, s.d. = 0.20, range: 1.25–1.90) with scores equal to or below 2 on Brysbaert *et al.*'s [42] concreteness scores to ensure that these items were unequivocally abstract and maximally contrasted with the concrete concepts (*n* = 15; moment, idea, hope, future, luck, value, peace, democracy, principle, imagination, concept, fantasy, ideal, perfection and beauty). We also selected highly concrete concepts (each selected concept had a concreteness score of 5) (*n* = 15; water, ball, tree, dog, camera, apple, grass, flower, snake, elephant, magazine, tomato, vase, jar and umbrella). Based on the norm database, spiritual concepts were on average more abstract (*M* = 2.53, s.d. = 0.99) than general concepts (*M* = 3.34, s.d. = 1.67). Frequency of the concrete concepts (*M* = 13 485, s.d. = 18 395, range: 1547–68 324) was lower than the frequency of the abstract concepts (*M* = 24 172, s.d. = 28 580, range: 1994–75 621).^5^

##### Measures

Participants rated each concept on sensory modalities and motor effectors, and self-identified spirituality (*r* = 0.85), religiosity (*r* = 0.83), and demographics, using the same measure as in Study 1.

##### Vaccination scepticism

We included a measure of vaccination scepticism (*α* = 0.80 [49]) that included five items (e.g. ‘I believe that vaccines are a safe and reliable way to help avert the spread of preventable diseases’ or ‘The risk of vaccinations to maim and kill children outweighs their health benefits’). Participants responded on a seven-point scale (1 = *strongly disagree*, 7 = *strongly agree*). The scores were recoded, such that high ratings indicated stronger scepticism.

### 6.2. Results

Zero-order correlations are presented in table 4. As in Study 1, we estimated a mixed-effects model but added an interaction between spirituality, concreteness and concept type: lmer(sensorimotor rating ∼ spirituality × concept concreteness × concept type + category + religiosity + conservatism + age + gender + (1 | participant) + (1 | concept). The results are presented in table 5 (also see figure 2).

**Table 4.** Zero-order correlations between beliefs and sensorimotor associations (Study 2).

	1	2	3
1. spirituality			
2. religiosity	0.65**		
	[0.51, 0.75]		
3. conservatism	0.18	0.19	
	[−0.02, 0.37]	[−0.01, 0.37]	
4. sensorimotor associations	0.20*	0.13	−0.05
	[0.00, 0.39]	[−0.07, 0.33]	[−0.25, 0.15]

**p < 0.001, *p < 0.05.

**Table 5.** Predictors of sensorimotor associations (Study 2).

	sensorimotor associations		
predictors	*β*	95% CI	*p*
spirituality	0.08	−0.01 to 0.17	0.076
concept concreteness	0.08	−0.00 to 0.16	0.052
concept type (general: 0, spiritual: 1)	0.10	0.03 to 0.17	0.006
category (motor: 0, sensory: 1)	0.01	0.01 to 0.02	0.001
conservatism	−0.04	−0.11 to 0.03	0.208
religiosity	0.01	−0.08 to 0.09	0.900
age	−0.01	−0.08 to 0.06	0.714
gender (women: 1)	0.09	0.02 to 0.16	0.010
spirituality × concept concreteness	−0.05	−0.05 to -0.04	0.001
spirituality × concept type	0.01	0.01 to 0.02	0.001
concept concreteness × concept type	0.06	−0.02 to 0.14	0.119
spirituality × concept concreteness × concept type	−0.01	−0.02 to 0.00	0.006
**random effects**			
*σ*^2^	0.79		
*τ*_00 ps_	0.11		
*τ*_00 item_	0.07		
ICC	0.19		
*n*_ps_	96		
*n*_item_	60		
observations	63 360		
marginal *R*^2^/conditional *R*^2^	0.028/0.210

**Figure 2.**
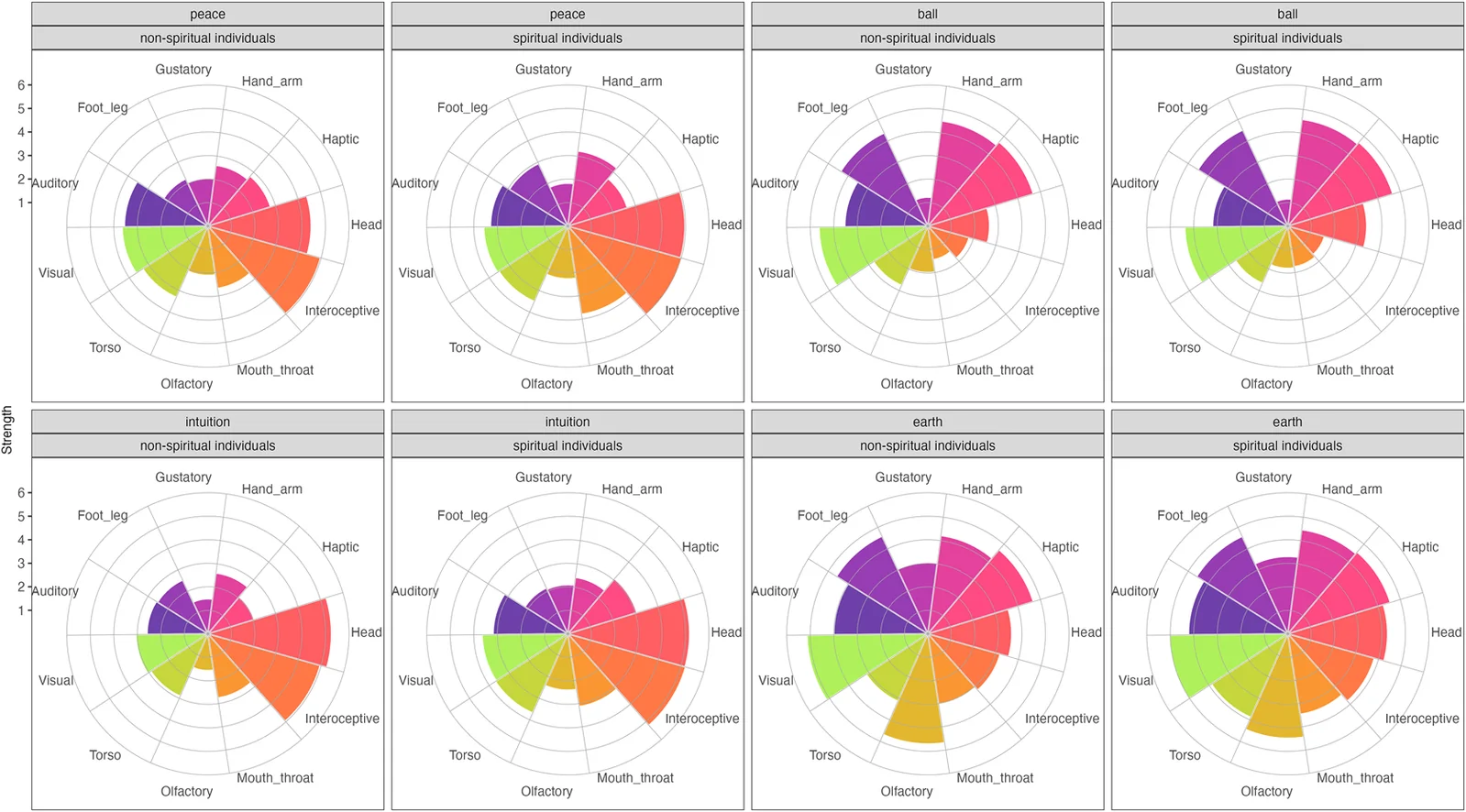
Sensorimotor strength for general abstract (peace), spiritual abstract (intuition), general concrete (ball) and spiritual concrete (earth) concepts for spiritual and non-spiritual individuals (median split) in Study 2.

#### 6.1.3. Confirmatory analyses

The predicted main effect of spirituality was not significant, but we found a three-way interaction between spirituality, concept concreteness and concept type. Simple slopes indicated that when concepts were highly abstract (low concreteness: −1s.d.) spirituality predicted greater sensorimotor associations for both spiritual (*β* = 0.15, 95% CI [0.06, 0.24], s.e. = 0.05, *t* = 3.28, *p* < 0.001) and general concepts (*β* = 0.13, 95% CI [0.04, 0.22], s.e. = 0.05, *t* = 2.75, *p <* 0.006). Trend analyses indicated that these slopes were significantly different from each other, estimate = 0.05, s.e. *=* 0.01, *z* = 4.83, *p* < 0.001. For highly concrete concepts (+1s.d.), the effect of spirituality was not significant for spiritual (*β* = 0.04, 95% CI [−0.05, 0.13], s.e. = 0.05, *t* = 0.81, *p* < 0.429) or general concepts (*β* = 0.04, 95% CI [−0.05, 0.13], s.e. = 0.05, *t* = 0.78, *p* < 0.437).^6^

We also found a two-way interaction between spirituality and concept concreteness, such that spirituality predicted greater sensorimotor associations for highly abstract concepts (*β* = 0.13, 95% CI [0.04, 0.22], s.e. = 0.05, *t* = 2.75, *p =* 0.010) but not for moderate (*β* = 0.08, 95% CI [0.001, 0.17], s.e. = 0.05, *t* = 1.77, *p =* 0.081) and highly concrete concepts (*β* = 0.04, 95% CI [0.05, 0.13], s.e. = 0.05, *t* = 0.78, *p =* 0.441). There was also an interaction between spirituality and concept type, with spirituality predicting stronger associations for general concepts (*β* = 0.09, 95% CI [0.001, 0.18], s.e. = 0.05, *t* = 2.06, *p =* 0.040), but not for spiritual concepts (*β* = 0.07, 95% CI [0.001, 0.16], s.e. = 0.05, *t* = 1.48, *p =* 0.144).

Finally, we found a main effect of concept type, with spiritual concepts being associated with greater sensorimotor associations than general concepts, and a main effect of sensorimotor category – sensory associations were stronger than motor associations.

#### 6.1.4. Exploratory analyses

Next, we tested whether spirituality interacted with sensorimotor associations in predicting greater vaccination scepticism. We computed mean sensorimotor association rating per participant and sensorimotor category. Subsequently, we estimated a regression model, with vaccination scepticism as the outcome. Spirituality and sensorimotor ratings were entered as predictors, controlling for sensorimotor category. We also included an interaction term between spirituality and sensorimotor ratings. The results are presented in table 6. The main effects of spirituality and sensorimotor associations were not significant. Yet, the spirituality by sensorimotor association interaction was significant. Simple slopes indicated that spirituality significantly predicted vaccination scepticism when sensorimotor associations were strong (+1s.d.), *β* = 0.22, 95% CI [0.04, 0.40], s.e. = 0.09, *t* = 2.42, *p =* 0.022. The other slopes were not significant (at the mean: *β* = 0.02, 95% CI [−0.12, 0.17], s.e. = 0.08, *t* = 0.32, *p =* 0.754, and at −1s.d.: *β* = *−*0.17, 95% CI [−0.40, 0.06], s.e. = 0.11, *t* = −1.49, *p =* 0.140).

**Table 6.** Spirituality and sensorimotor associations predicting vaccination scepticism (Study 2).

	vaccination scepticism		
predictors	*β*	95% CI	*p*
sensorimotor associations	−0.04	−0.19 to 0.10	0.571
spirituality	0.02	−0.13 to 0.17	0.759
category (motor: 0, sensory: 1)	0.03	−0.25 to 0.31	0.842
sensorimotor associations × spirituality	0.19	0.05 to 0.34	0.007
observations	192		
*R*^2^/*R*^2^ adjusted	0.045/0.024		

#### 6.1.5. Summary

Overall, although the main effect of spirituality was not significant, spirituality significantly predicted stronger sensorimotor associations for highly abstract concepts regardless of concept type (i.e. spiritual vs. general). In the exploratory analysis, we also found that spirituality interacted with sensorimotor associations in predicting stronger vaccination scepticism, with spirituality being particularly linked to scepticism when sensorimotor associations were high. In Study 3, we aimed to replicate these effects in another sample of participants.

## 7. Study 3: Replication with spiritual concepts

In Study 3, we aimed to replicate the findings from Study 2 in another sample of participants, again using spiritual concepts as in Study 1 and assessing whether sensorimotor grounding predicts greater vaccination rejection among spiritual individuals. All hypotheses, materials and analysis plans were pre-registered on the Open Science Framework: https://doi.org/10.17605/OSF.IO/UG8JH.

### 7.1. Methods

#### 7.1.1. Participants

We recruited 154 participants through a Cardiff University participant pool based on our preregistration plan and the previously conducted power analysis. We used a different university pool than in Study 2. We excluded participants who failed at least one attention check. The total sample size was 139 participants (see table 1 for demographic information). Participants received course credits as compensation for a 60 min study. The study received Cardiff University ethics approval: EC.25.02.11.7160.

#### 7.1.2. Material

We selected the same spiritual-concrete and spiritual-abstract concepts as in Study 1 (*n* = 30). Owing to resource constraints, we deviated from the preregistration plan and tested only spiritual rather than spiritual and general concepts. We measured spirituality (*r* = 0.80), religiosity (*r* = 0.91), demographics and vaccination scepticism using the same measure as in Study 2 (*α* = 0.84). Owing to a coding mistake, one item (‘The risk of vaccinations to maim and kill children outweighs their health benefits’) was omitted, and item ‘Vaccines are thoroughly tested in the laboratory and wouldn't be made available to the public unless it was known that they are safe’ was presented twice. The two items were highly correlated, *r* = 0.89. We computed means across all items (including the duplicated item). We also measured other variables that are not reported in this paper: belief in science, beliefs in universalism and intuitive epistemology and the naturalness bias.

### 7.2. Results

As in previous studies, we first computed zero-order correlations (table 7) and estimated a mixed-effects model. The structure of the model was the same as in Study 1. We found that spirituality significantly predicted stronger sensorimotor ratings, and this effect was moderated by concept concreteness (table 8).

**Table 7.** Zero-order correlations between beliefs and sensorimotor associations (Study 3).

	1	2	3
1. spirituality			
2. religiosity	0.56**		
	[0.43, 0.66]		
3. conservatism	0.09	0.25**	
	[−0.08, 0.25]	[0.08, 0.40]	
4. sensorimotor associations	0.41**	0.14	0.04
	[0.26, 0.54]	[−0.03, 0.30]	[−0.13, 0.21]

**p < 0.001, *p < 0.05.

**Table 8.** Predictors of sensorimotor associations (Study 3).

	sensorimotor associations		
predictors	*β*	95% CI	*p*
spirituality	0.23	0.14 to 0.31	0.001
concept concreteness	0.06	−0.05 to 0.18	0.283
category (motor: 0, sensory: 1)	0.01	0.00 to 0.02	0.045
religiosity	0.02	−0.05 to 0.10	0.548
conservatism	−0.07	−0.16 to 0.02	0.117
age	−0.06	−0.13 to 0.01	0.101
gender (1: women)	−0.01	−0.08 to 0.06	0.740
spirituality × concept concreteness	−0.02	−0.02 to -0.01	0.001
**random effects**			
*σ*^2^	0.68		
*τ*_00 ps_	0.17		
*τ*_00 item_	0.10		
ICC	0.29		
*n*_ps_	137		
*n*_item_	30		
observations	45 210		
marginal *R*^2^/conditional *R*^2^	0.045/0.318		

While spirituality was significantly associated with stronger sensorimotor associations at all levels of concept concreteness: low (−1s.d.), *β* = 0.24, 95% CI [0.16, 0.33], s.e. = 0.04, *t* = 5.62, *p* < 0.001, moderate (mean), *β* = 0.23, 95% CI [0.14, 0.31], s.e. = 0.04, *t* = 5.27, *p* < 0.001, and high (+1s.d.), *β* = 0.21, 95% CI [0.13, 0.30], s.e. = 0.04, *t* = 4.88, *p* < 0.001, the differences between slopes were significant, such that the spirituality-sensorimotor strength link was stronger for low than high concreteness, estimate = 0.03, s.e. *=* 0.01, *z* = 4.11, *p* < 0.001, and low and moderate concreteness, estimate = 0.02, s.e. *=* 0.01, *z* = 4.11, *p* < 0.001. The difference between moderate and high was also significant, estimate = 0.02, s.e. *=* 0.01, *z* = 4.11, *p* < 0.001. We also found a small main effect of sensorimotor category, with stronger associations for sensory as opposed to motor modalities.

We subsequently tested whether spirituality predicted stronger vaccination scepticism and whether this effect was moderated by sensorimotor associations. We computed the same model as in Study 2 (table 9). In partial support of our hypothesis, we found that spirituality predicted stronger vaccination scepticism, yet this effect was not moderated by sensorimotor strength. We aimed to follow up on these findings in another high-powered replication by recruiting the general public.

**Table 9.** Spirituality and sensorimotor associations predicting vaccination scepticism (Study 3).

	vaccination scepticism		
predictors	*β*	95% CI	*p*
sensorimotor associations	0.07	−0.06 to 0.20	0.302
spirituality	0.16	0.03 to 0.29	0.014
category (motor: 0, sensory: 1)	−0.00	−0.12 to 0.12	0.992
sensorimotor associations × spirituality	−0.06	−0.16 to 0.04	0.319
observations	274		
*R*^2^/*R*^2^ adjusted	0.040/0.026		

## 8. Study 4: High-powered replication

In Study 4, we aimed at replicating our previous findings by using a smaller number of concepts, but a larger sample size. The hypotheses were the same as in Studies 2 and 3.

### 8.1. Methods

#### 8.1.1. Participants

We recruited 351 British residents through the online recruitment platform Prolific to provide a high-powered replication of the previous findings. Owing to a coding error, 46 participants were not presented with the attention check item. All other participants passed the attention check, so we did not exclude any participants (see table 1 for demographic information). Participants received £1.30 for participation in a 10-min study. The study received Gettysburg College ethics approval: 100925BM.

#### 8.1.2. Material

We selected six spirituality-related concepts based on the variability in how strongly their sensorimotor strength correlated with spirituality (derived from Study 1), with three items relatively more concrete: weak correlations: nature (*r* = 0.21; concrete) and connection (*r* = 0.26; abstract), and moderate-to-strong correlations: body (*r* = 0.34; concrete) and universalism (*r* = 0.34; abstract), universe (*r* = 0.35; concrete) and soul (*r* = 0.46; abstract). We included the same measures as in the previous studies: spirituality (*r* = 0.89), religiosity (*r* = 0.91), demographics and vaccination scepticism (*α* = 0.90).^7^

### 8.2. Results

Zero-order correlations are presented in table 10. Consistent with previous findings, spirituality was associated with stronger sensorimotor ratings. This effect was moderated by concept concreteness (table 11).

**Table 10.** Zero-order correlations between beliefs and sensorimotor associations (Study 4).

	1	2	3
1. spirituality			
2. religiosity	0.62**		
	[0.55, 0.68]		
3. conservatism	0.09	0.11*	
	[−0.02, 0.19]	[0.00, 0.21]	
4. sensorimotor associations	0.40**	0.19**	0.02
	[0.30, 0.48]	[0.09, 0.29]	[−0.08, 0.13]

**p < 0.001, *p < 0.05.

**Table 11.** Predictors of sensorimotor associations (Study 4).

	sensorimotor associations		
predictors	*β*	95% CI	*p*
spirituality	0.23	0.17–0.30	0.001
concept concreteness	0.25	0.03–0.47	0.029
category (motor: 0, sensory: 1)	0.06	0.05–0.07	0.001
conservatism	−0.00	−0.05 to 0.05	0.985
religiosity	−0.04	−0.11 to 0.02	0.174
age	−0.03	−0.08 to 0.02	0.229
gender (1: women)	−0.01	−0.06 to 0.04	0.609
spirituality × concept concreteness	−0.02	−0.03 to -0.01	0.001
**random effects**			
*σ*^2^	0.60		
*τ*_00 ps_	0.21		
*τ*_00 item_	0.08		
ICC	0.33		
*n*_ps_	351		
*n*_item_	6		
observations	21 641		
marginal *R*^2^/conditional *R*^2^	0.110/0.400		

Again, the association between spirituality and sensorimotor associations was the strongest when concept abstractness was high: low (−1s.d.), *β* = 0.25, 95% CI [0.19, 0.32], s.e. = 0.03, *t* = 7.76, *p* < 0.001, compared to the mean, *β* = 0.23, 95% CI [0.17, 0.30], s.e. = 0.03, *t* = 7.23, *p* < 0.001, (estimate = 0.02, s.e. *=* 0.01, *z* = 3.83, *p* < 0.001), and compared to high concreteness (+1s.d.), *β* = 0.21, 95% CI [0.15, 0.28], s.e. = 0.03, *t* = 6.51, *p* < 0.001 (estimate = 0.04, s.e. *=* 0.01, z = 3.83, *p* < 0.001). The difference in slopes between mean and high (+1s.d.) concept concreteness was also significant (estimate = 0.02, s.e. *=* 0.01, *z* = 3.83, *p* < 0.001).

We also found a main effect of sensorimotor category, such that the associations were generally greater for sensory modalities compared to motor effectors, and a main effect of concept concreteness, with higher concreteness predicting greater sensorimotor associations.

Finally, we tested whether spirituality involved stronger vaccination scepticism and whether this was moderated by sensorimotor associations (table 12). Although spirituality predicted stronger vaccination scepticism in partial support of our hypothesis, the spirituality-by-sensorimotor-associations interaction was not significant. We additionally found a main effect of sensorimotor associations, whereby stronger associations predicted greater vaccination scepticism.

**Table 12.** Spirituality and sensorimotor associations predicting vaccination scepticism (Study 4).

	vaccination scepticism		
predictors	*β*	predictors	*β*
sensorimotor associations	0.08	0.01–0.16	0.031
spirituality	0.35	0.27–0.42	0.001
category (motor: 0, sensory: 1)	−0.01	−0.08 to 0.06	0.795
sensorimotor associations × spirituality	0.01	−0.06 to 0.07	0.817
observations	702		
*R*^2^/*R*^2^ adjusted	0.152/0.147		

Overall, we replicated the findings from Studies 1–3 regarding the link between spirituality and sensorimotor features. Yet, the results concerning vaccination scepticism were not consistent with the previous studies. Future studies should more closely assess how sensorimotor grounding contributes to science rejection.

## 9. General discussion

Recent theoretical perspectives on abstract concepts increasingly emphasize the multidimensionality of abstract concepts and the importance of individual experience in conceptual representations [3,5,6]. Indeed, the meaning of abstract concepts is flexible, influenced by socio-cultural context and social metacognition, and it varies across people with diverse sensory experiences [3,5,6,13]. In the current paper, we aimed to understand whether beliefs about ontological meaning represent an important factor that moderates conscious sensorimotor associations with abstract concepts. We hypothesized that spirituality, which focuses on an experiential approach to understanding reality, would contribute to stronger attendance to sensorimotor features in abstract compared to concrete concepts [3,6,13]. Importantly, as spirituality is conceptually related to religiosity, we simultaneously assessed the predictive power of spirituality and religiosity in sensorimotor associations.

We tested the predictions across four studies, where we asked participants to indicate how strongly they experienced a series of spiritual and general concepts that varied in abstractness with perceptual modalities and motor effectors. Across all studies, spirituality predicted greater sensorimotor strength especially for highly abstract compared to concrete concepts, when controlling for religiosity, the domain (sensory vs. motor), political ideology and demographics. Across all studies, the main effect of religiosity was not significant. In addition, in Study 2, which assessed a large pool of general and spiritual concepts (*N* = 60), we further found that spirituality involved especially strong sensorimotor associations for general and spiritual concepts that were highly abstract. Finally, consistent with the notion that the meaning of highly concrete concepts is generally less subjective and varies less across people [3], spirituality did not significantly predict sensorimotor associations of highly concrete spiritual and general concepts.

Finally, as spirituality predicts stronger science rejection [11,32,38], we also assessed whether sensorimotor grounding functions as a reinforcing mechanism for experiential epistemology, contributing to increased science rejection among spiritual individuals. Yet, the evidence for these associations was mixed, and this question requires further investigation.

### 9.1. Spirituality guides abstract representations

Current perspectives on cognition suggest that contextual features, concept types and different sensory or motor experiences guide how strongly abstract conceptual knowledge is grounded in sensorimotor dimensions [3,6,7,15]. Although evidence is still limited, the literature increasingly acknowledges the importance of individual differences in conceptual representations [5]. Indeed, previous studies found that sensorimotor grounding of abstract concepts depends on individual differences in demographics, socio-cultural influences and diversity of sensory experiences, including those of blind or deaf individuals [26–28,50,51]. Our findings directly extend this literature by demonstrating that belief systems are another important dimension in conceptual associations. One possibility is that higher-order cognition motivated by pursuit of ontological meaning through an experiential epistemology guides lower-level embodied conceptual representations. On the other hand, a reversed pattern is also plausible: individuals who more strongly attend to sensorimotor experiences while acquiring concepts may adopt spiritual belief systems. Overall, our findings contribute to literature on abstract concepts as well as spiritual belief systems.

First, in illustrating the importance of belief systems, our evidence suggests that the nature of concept, type of perceptual or motor experience and contextual or physical features of the environment do not fully account for associations in abstract concepts. Although previously it was assumed that abstract concepts are less linked to sensorimotor experiences on average (e.g. as demonstrated by how highly abstract spiritual and philosophical concepts are weakly associated with sensorimotor dimensions [7]), other studies showed that concrete as well as some abstract categories involve equally strong sensorimotor links in multiple perceptual and motor domains [13]. These findings imply that the concrete-abstract dichotomy is not diagnostic when determining the representation of concepts [5]. Consistent with this literature, our evidence further suggests that mentally conscious thoughts about abstract concepts are guided by an interaction between conceptual characteristics, that is concept concreteness, and belief systems. As such, sensorimotor features are present in abstract thoughts depending on individualized ways of understanding reality. It is therefore likely that, beyond conceptual characteristics, language and social interactions with others, motivational approaches to meaning have important implications also for conceptual representations and sensorimotor grounding.

Second, our findings provide empirical evidence for the theoretical and historical insights concerning spiritual beliefs. Spiritual beliefs are theoretically defined as a unique way of knowing focused on experiential approaches to knowledge that are distinct from scientific reason and religious faith [29]. In that sense, spiritual meaning cannot be independently verified (unlike scientific reason) or communicated to another person (unlike scientific reason and religious faith), as it is highly individualized, personal and shaped by experience [29,30]. Our findings support the view that spirituality is experiential which is manifested by greater links between bodily experiences and abstract phenomena in lower-order cognition.

Importantly, religiosity did not significantly contribute to sensorimotor associations, despite religious prayer practices often being linked to embodied dimensions, such as postures [52]. It is worth mentioning, however, that zero-order correlations in Studies 1 and 4 (but not in Studies 2 and 3) demonstrate that religiosity is also significantly linked to sensorimotor strength (Study 1: *r* = 0.28; Study 4: *r* = 0.19), although to a lower extent than spirituality (Study 1: *r* = 0.41; Study 4: *r* = 0.40). While religiosity has not been linked to experiential approaches to truth, it does involve stronger reliance on intuition, while religious practice can also lead to spiritual experiences [53,54]. Nevertheless, these zero-order correlations are explained by spirituality in Studies 1 and 4, as religiosity does not predict sensorimotor strength when spirituality is controlled for. This suggests that although spirituality conceptually overlaps with religiosity, spirituality has uniquely strong links with embodiment.

### 9.2. Potential mechanisms

Although we did not investigate mechanisms involved, the links between spiritual beliefs and sensorimotor features may potentially arise in response to an interaction between a need to assign greater personal significance to bodily experience and greater focus or attention to these experiences. Recent work suggests that supernatural beliefs likely occur in response to anomalous or ambiguous sensory experiences. When causal factors leading to such experiences are not readily accessible and the experiences are particularly emotional or aversive, supernatural beliefs are used to account for them [55]. In turn, these beliefs are retained among those who are prone to cognitive biases, such as teleological thinking whereby experiences are explained as if designed for a purpose [56]. It is therefore possible that spiritual individuals, who have increased tendencies to think teleologically and intuitively, also pay particular attention to their bodily experiences which in turn helps the formation of sensorimotor associations with abstract thought. In turn, spirituality-embodiment links may be further reinforced and maintained by cultural spiritual practices, involving perceptual and motor activities, such as yoga or meditation [11,57]. Indeed, previous work suggests that stronger sensory and motor activation in the brain (during action perception) is associated with having training or expertise compared to having no training in specific activities or movements like professional dancing [58–61]. Future research should directly examine whether sensorimotor associations are explained by greater attention to bodily sensations and a dispositional tendency for teleological thinking among spiritual individuals.

### 9.3. Implications for science rejection

While in all studies we found that spirituality was associated with vaccine scepticism, supporting previous findings in the literature, we found mixed evidence for the effect of sensorimotor associations [31,38]. Although the results in Study 2 supported our hypothesis, as spirituality predicted stronger vaccination rejection especially when sensorimotor associations were strong, Study 4 demonstrated that sensorimotor grounding and spirituality contributed independently to stronger vaccination rejection. These results did not replicate in Study 3, where sensorimotor associations did not significantly predict vaccination scepticism. Potentially, these associations contribute directly to low belief in science as a way of knowing (i.e. epistemological conflict between external scientific knowledge and inner spiritual knowledge), rather than being directly involved in vaccination rejection. Nevertheless, this evidence hints on the importance of conceptual associations for higher-order attitudes to different epistemologies. Potentially increased grounding of the meaning of abstract concepts in perception and action creates metaphoric links between bodily experiences and perceptions of ontological meaning [16]. When these perceptions of valued meaning conflict with scientific findings, knowledge that feels meaningful is favoured over objective evidence by spiritual individuals. This should be directly examined in future studies.

### 9.4. Limitations

While we found that spirituality is uniquely linked to greater sensorimotor features in highly abstract concepts, our findings are based on studies that largely assessed sensorimotor associations of spirituality-related concepts rather than non-spiritual concepts that we termed general concepts (although spirituality also predicted stronger sensorimotor grounding of highly abstract general concepts in Study 2). It is important to note that while the preselected items were categorized as relevant and irrelevant to spirituality based on previous classifications (e.g. [7]), this distinction has not been objectively validated. First, this distinction may vary based on individual differences in spirituality; for example, the concept of peace might be seen as more spiritual to spiritual than non-spiritual individuals. Second, general concepts had more extreme concreteness and abstractness scores than the spiritual concepts. It is possible that individual differences in spirituality are not only associated with conscious attention to sensorimotor features, but that the observed differences between spiritual and non‑spiritual concepts were partly driven by variability in the degree of agreement for those concepts. The present findings therefore suggest that future research should directly examine whether levels of agreement differ in judgements of the concreteness of spiritual versus general concepts and whether such agreement is modulated by individual differences in spirituality. It is also important to acknowledge that the number of items used in the present studies was limited (especially in Study 4) and may not be representative of a broader conceptual lexicon. Therefore, to establish whether the reported effects generalize to a broader set of concepts, it is necessary to examine a larger stimuli pool in future research.

Finally, we note that our operationalization of spirituality involved spiritual identity (Studies 1–4) and post-Christian beliefs about epistemology (Study 1). We did not investigate the relationship between spiritual practices or rituals and sensorimotor associations. It is possible that spiritual individuals who engage in specific rituals might show especially strong motor compared to sensory associations with abstract concepts.

## 10. Conclusion

Cultural beliefs related to spirituality can satisfy a fundamental human need for understanding reality by offering a promise that ontological meaning can be found through inner experiences [30]. We therefore aimed to examine whether commitment to spiritual beliefs moderates the extent to which abstract concepts are linked to sensorimotor experiences. Across four studies, we found that spirituality involves stronger sensorimotor associations in abstract compared to concrete concepts, with spiritual individuals likely feeling meaning through linking abstract thought with their unique sensorimotor experiences. Overall, these findings demonstrate that attitudes towards ontological meaning guide thoughts about abstract concepts.

## Supplementary Material



## Data Availability

The dataset supporting this study is publicly available at https://osf.io/v5mc4. Supplementary material is available online [62].

## Footnotes

^1^
Note, in an open-ended question, participants indicated that they considered connection and energy as abstract concepts referring to connecting to others and good energy/vibes, respectively.

^2^
Owing to a coding error during the selection process, we initially classified holism as a concrete concept, but its rating indicated it was abstract.

^3^
Across all studies, we included exploratory measures, such as attitudes towards subjective epistemology. These are beyond the scope of the present paper.

^4^
The results were replicated in an exploratory analysis where we used the post-Christian spirituality scale instead of self-identified spirituality; main effect of spirituality *β =* 0.28, 95% CI [0.18, 0.37], *p <* 0.001; interaction: *β =* −0.02, 95% CI [−0.03, −0.01], *p <* 0.001.

^5^
For exploratory purposes, we also included concepts based on previous research measuring spiritual epistemology [47,48] and these included consciousness, harmony, high, life, order and spirit, and those relevant to vaccination: vaccine, virus, injection, immunity and antibodies. The results below are reported based on the core 60 concepts, but including the additional concepts did not change the results.

^6^
As the concreteness scores for general concepts did not follow a normal distribution and no general concepts fell on the mean, we limited the simple slopes analysis to −1 s.d. and +1 s.d.

^7^
We also measured other variables that were collected as part of another project and are not reported in this paper: the naturalness bias/conspiracy beliefs.
