# Supplementary material for: Can you feel meaning? Spirituality involves stronger sensorimotor associations with abstract concepts
Source: R Soc Open Sci. 2026 Oct 28;13(10):260997. doi: 10.1098/rsos.260997 (PMC13714391; doi:10.1098/rsos.260997)
Supplement: Supplementary file 1 [file rsos.260997.zip › rsos.260997/rsos260997_si_001.docx]

**Supplemental Materials**

**Robustness Analyses**

To rule out the possibility that religiosity also involves stronger sensorimotor ratings for highly abstract concepts, we conducted additional tests. In each study, we introduced an interaction term between religiosity (instead of spirituality) and concreteness scores: lmer(sensorimotor rating ~ religiosity*concept concreteness + category + spirituality + conservatism + age + gender + (1 | participant) + (1 | concept)

**Study 1**

Religiosity significantly interacted with concreteness scores, *β* = -.03, 95% CI [-.03, -.02], *p* < .001. Yet, the simple slopes analysis indicated that none of the slopes were significant, suggesting that religiosity did not predict stronger sensorimotor associations at different concreteness levels: -1SD: *β* = .06, 95% CI [-.06, .17], *SE* = .06, *t* = 0.96, *p* = .338; Mean: *β* = .03, 95% CI [-.08, .14], *SE* = .06, *t* = 0.50, *p* = .618; +1SD: *β* = .001, 95% CI [-.11, .11], *SE* = .06, *t* = 0.04, *p* = .970.

**Study 2**

The three-way interaction (religiosity, concept concreteness, concept type [general vs. spiritual] was not significant, *β* = -.01, 95% CI [-.01, .01], *p* = .456.

**Study 3**

As in Study 1, we found that the religiosity by concept concreteness was significant, *β* = -.02, 95% CI [-.03, -.02], *p* < .001. However, the religiosity slopes at -1SD (*β* = -.05, 95% CI [-.14, .04], *SE* = .05, *t* = -1.05, *p* = .294) and at the mean (*β* = -.07, 95% CI [-.16, .02], *SE* = .05, *t* = -1.57, *p* = .119) were not significant. The only significant slope was at +1SD, with lower religiosity linked to stronger sensorimotor associations for highly concrete concepts (*β* = -.09, 95% CI [-.18, -.01], *SE* = .05, *t* = -2.07, *p* = .040).

**Study 4**

The interaction between religiosity and concept concreteness was not significant, *β* = -.01, 95% CI [-.02, .01], *p* = .061.

**Frequency Analysis**

**Supplemental Table 1**

*Predictors of Sensorimotor Associations Controlling for Word Frequency (Study 1).*

|  | **Sensorimotor Associations** | | |
| --- | --- | --- | --- |
| *Predictors* | *β* | 95% *CI* | *p* |
| Spirituality | 0.22 | 0.11 – 0.32 | .001* |
| Concept Concreteness | -0.00 | -0.09 – 0.08 | .943 |
| Word Frequency | 0.17 | 0.09 – 0.26 | .001* |
| Category (Motor: 0, Sensory: 1) | 0.03 | -0.08 – 0.14 | .616 |
| Religiosity | 0.00 | -0.01 – 0.01 | .957 |
| Conservatism | -0.04 | -0.13 – 0.05 | .343 |
| Age | 0.03 | -0.05 – 0.11 | .434 |
| Gender (1: Women) | 0.07 | -0.01 – 0.15 | .085 |
| Spirituality × Concept Concreteness | -0.03 | -0.03 – -0.02 | .001* |
| **Random Effects** | | | |
| σ^2^ | 0.64 | | |
| τ_00_ _ps_ | 0.23 | | |
| τ_00_ _item_ | 0.05 | | |
| ICC | 0.30 | | |
| N _ps_ | 141 | | |
| N _item_ | 30 | | |
| Observations | 45040 | | |
| Marginal R^2^ / Conditional R^2^ | 0.087 / 0.365 | | |

**Supplemental Table 2**

*Predictors of Sensorimotor Associations Controlling for Word Frequency (Study 2).*

|  | **Sensorimotor Associations** | | |
| --- | --- | --- | --- |
| *Predictors* | *β* | *95% CI* | *p* |
| Spirituality | 0.08 | -0.01 – 0.17 | .079 |
| Concept Concreteness | 0.07 | -0.00 – 0.15 | .065 |
| Concept Type (General: 0, Spiritual: 1) | 0.14 | 0.07 – 0.21 | .001* |
| Word Frequency | 0.11 | 0.04 – 0.18 | .001* |
| Category (Motor: 0, Sensory: 1) | 0.02 | -0.16 – 0.19 | .860 |
| Conservatism | -0.05 | -0.12 – 0.02 | .202 |
| Religiosity | 0.01 | -0.08 – 0.10 | .888 |
| Age | -0.01 | -0.08 – 0.06 | .704 |
| Gender (Women: 1) | 0.09 | 0.02 – 0.16 | .011* |
| Spirituality × Concept Concreteness | -0.05 | -0.05 – -0.04 | .001* |
| Spirituality × Concept Type | 0.01 | 0.01 – 0.02 | .001* |
| Concept Concreteness × Concept Type | 0.02 | -0.05 – 0.10 | .533 |
| Spirituality × Concept Concreteness  × Concept Type | -0.01 | -0.02 – -0.00 | .003* |
| **Random Effects** | | | |
| σ^2^ | 0.71 | | |
| τ_00_ _ps_ | 0.11 | | |
| τ_00_ _item_ | 0.06 | | |
| τ_00_ _modality_ | 0.08 | | |
| ICC | 0.27 | | |
| N _ps_ | 96 | | |
| N _item_ | 60 | | |
| N _modality_ | 11 | | |
| Observations | 61248 | | |
| Marginal R^2^ / Conditional R^2^ | 0.038 / 0.296 | | |

*Note*: Simple Slopes for the Spirituality × Concept Concreteness × Concept Type interaction (Study 2): Slope for spirituality predicting sensorimotor associations for concepts with low concreteness (-1SD): spiritual concepts, *β = .*15, 95% CI [.06, .24], *SE* = .05, *t =* 3.28, *p < .*001, and general concepts, *β = .*12, 95% CI [.03, .22], *SE* = .05, *t =* 2.70, p < .008; at the mean: spiritual concepts: *β = .*09, 95% CI [.01, .24], *SE* = .05, *t =* 1.99, *p = .*050, and general concepts: *β = .*07, 95% CI [-.02, .16], *SE* = .05, *t =* 1.43, *p = .*150; and high concreteness (+1SD): spiritual concepts, *β = .*03, 95% CI [-.06, .13], *SE* = .05, *t = .*74, *p = .*463; general concepts: *β = .*03, 95% CI [-.06, .12], *SE* = .05, *t =* 0.71, *p = .*482.

**Supplemental Table 3**

*Predictors of Sensorimotor Associations Controlling for Word Frequency (Study 3).*

|  | **Sensorimotor Associations** | | |
| --- | --- | --- | --- |
| *Predictors* | *β* | *95% CI* | *p* |
| Spirituality | 0.23 | 0.14 – 0.31 | .001* |
| Concept Concreteness | -0.01 | -0.10 – 0.09 | .915 |
| Word Frequency | 0.21 | 0.12 – 0.31 | .001* |
| Category (Motor: 0, Sensory: 1) | 0.01 | 0.00 – 0.02 | .045* |
| Conservatism | 0.02 | -0.05 – 0.10 | 0.548 |
| Religiosity | -0.07 | -0.16 – 0.02 | 0.117 |
| Age | -0.06 | -0.13 – 0.01 | 0.100 |
| Gender (1: Women) | -0.01 | -0.08 – 0.06 | 0.740 |
| Spirituality × Concept Concreteness | -0.02 | -0.02 – -0.01 | .001* |
| **Random Effects** | | | |
| σ^2^ | 0.68 | | |
| τ_00_ _ps_ | 0.17 | | |
| τ_00_ _item_ | 0.06 | | |
| ICC | 0.25 | | |
| N _ps_ | 137 | | |
| N _item_ | 30 | | |
| Observations | 45210 | | |
| Marginal R^2^ / Conditional R^2^ | 0.086 / 0.318 | | |

**Supplemental Table 4**

*Predictors of Sensorimotor Associations Controlling for Word Frequency (Study 4).*

|  | **Sensorimotor Associations** | | |
| --- | --- | --- | --- |
| *Predictors* | *β* | 95*% CI* | *p* |
| Spirituality | 0.23 | 0.17 – 0.30 | .001* |
| Concept Concreteness | 0.04 | -0.29 – 0.37 | .822 |
| Word Frequency | 0.26 | -0.07 – 0.59 | .122 |
| Category (Motor: 0, Sensory: 1) | 0.06 | 0.05 – 0.07 | .001* |
| Conservatism | -0.00 | -0.05 – 0.05 | .985 |
| Religiosity | -0.04 | -0.11 – 0.02 | .175 |
| Age | -0.03 | -0.08 – 0.02 | .229 |
| Gender (1: Women) | -0.01 | -0.06 – 0.04 | .609 |
| Spirituality × Concept Concreteness | -0.02 | -0.03 – -0.01 | .001* |
| **Random Effects** | | | |
| σ^2^ | 0.60 | | |
| τ_00_ _ps_ | 0.21 | | |
| τ_00_ _item_ | 0.06 | | |
| ICC | 0.31 | | |
| N _ps_ | 351 | | |
| N _item_ | 6 | | |
| Observations | 21641 | | |
| Marginal R^2^ / Conditional R^2^ | 0.132 / 0.400 | | |

**Modality Analysis**

**Supplemental Table 5**

*Predictors of Sensory Associations Controlling for Modalities (Reference Category: Interoception) (Study 1).*

|  | **Sensory Associations** | | |
| --- | --- | --- | --- |
| *Predictors* | *β* | 95*% CI* | *p* |
| Spirituality | 0.20 | 0.09 – 0.31 | **.001** |
| Concept Concreteness | 0.04 | -0.06 – 0.15 | .422 |
| Modality: Touch | -0.29 | -0.32 – -0.26 | **.001** |
| Modality: Hearing | -0.28 | -0.31 – -0.25 | **.001** |
| Modality: Seeing | -0.12 | -0.15 – -0.09 | **.001** |
| Modality: Smelling | -0.63 | -0.67 – -0.60 | **.001** |
| Modality: Tasting | -0.73 | -0.76 – -0.70 | **.001** |
| Religiosity | 0.02 | -0.09 – 0.14 | .710 |
| Conservatism | -0.04 | -0.14 – 0.05 | .338 |
| Age | 0.04 | -0.04 – 0.12 | .351 |
| Gender (1: Women) | 0.14 | -0.01 – 0.29 | .074 |
| Spirituality x Concept Concreteness | -0.03 | -0.04 – -0.02 | **.001*** |
| **Random Effects** | | | |
| σ^2^ | 0.55 | | |
| τ_00_ _ps_ | 0.25 | | |
| τ_00_ _item_ | 0.08 | | |
| ICC | 0.38 | | |
| N _ps_ | 141 | | |
| N _item_ | 30 | | |
| Observations | 24570 | | |
| Marginal R^2^ / Conditional R^2^ | 0.121 / 0.452 | | |

**Supplemental Table 6**

*Predictors of Motor Associations Controlling for Modalities (Reference Category: Head No Mouth) (Study 1).*

|  | **Motor Associations** | | |
| --- | --- | --- | --- |
| *Predictors* | *β* | 95*% CI* | *p* |
| Spirituality | 0.23 | 0.12 – 0.35 | **.001*** |
| Concept Concreteness | 0.06 | -0.04 – 0.17 | .227 |
| Modality: Hand/Arm | -0.43 | -0.46 – -0.40 | **.001*** |
| Modality: Foot/Leg | -0.55 | -0.58 – -0.52 | **.001*** |
| Modality: Torso | -0.47 | -0.50 – -0.44 | **.001*** |
| Modality: Mouth/Throat | -0.48 | -0.52 – -0.45 | **.001*** |
| Religiosity | 0.04 | -0.08 – 0.16 | .557 |
| Conservatism | -0.04 | -0.13 – 0.05 | .411 |
| Age | 0.02 | -0.06 – 0.11 | .606 |
| Gender (Women: 1) | 0.12 | -0.04 – 0.27 | .139 |
| Spirituality × Concept Concreteness | -0.02 | -0.03 – -0.01 | **.001*** |
| **Random Effects** | | | |
| σ^2^ | 0.55 | | |
| τ_00_ _ps_ | 0.26 | | |
| τ_00_ _item_ | 0.08 | | |
| ICC | 0.39 | | |
| N _ps_ | 141 | | |
| N _item_ | 30 | | |
| Observations | 20470 | | |
| Marginal R^2^ / Conditional R^2^ | 0.109 / 0.452 | | |

**Supplemental Table 7**

*Predictors of Sensory Associations Controlling for Modalities (Reference Category: Interoception) (Study 2).*

|  | **Sensory Associations** | | |
| --- | --- | --- | --- |
| *Predictors* | *β* | 95*% CI* | *p* |
| Spirituality | 0.05 | -0.04 – 0.14 | .245 |
| Concept Concreteness | 0.10 | 0.01 – 0.18 | **.027*** |
| Concept Type (General: 0, Spiritual: 1) | 0.06 | -0.01 – 0.14 | .106 |
| Modality: Touch | -0.09 | -0.12 – -0.06 | **.001*** |
| Modality: Hearing | -0.16 | -0.19 – -0.13 | **.001*** |
| Modality: Seeing | 0.27 | 0.24 – 0.30 | **.001*** |
| Modality: Smelling | -0.54 | -0.57 – -0.51 | **.001*** |
| Modality: Tasting | -0.70 | -0.73 – -0.67 | **.001*** |
| Religiosity | -0.00 | -0.09 – 0.08 | .928 |
| Conservatism | -0.02 | -0.09 – 0.05 | .598 |
| Age | -0.01 | -0.07 – 0.06 | .847 |
| Gender (Women: 1) | 0.18 | 0.01 – 0.34 | **.033*** |
| Spirituality × Concept Concreteness | -0.05 | -0.06 – -0.04 | **.001*** |
| Spirituality × Concept Type | 0.03 | 0.02 – 0.04 | **.001*** |
| Concept Concreteness × Concept Type | 0.04 | -0.05 – 0.13 | .363 |
| Spirituality × Concept Concreteness  × Concept Type | -0.03 | -0.04 – -0.02 | **.001*** |
| **Random Effects** | | | |
| σ^2^ | 0.70 | | |
| τ_00_ _ps_ | 0.11 | | |
| τ_00_ _item_ | 0.08 | | |
| ICC | 0.21 | | |
| N _ps_ | 96 | | |
| N _item_ | 60 | | |
| Observations | 34560 | | |
| Marginal R^2^ / Conditional R^2^ | 0.124 / 0.310 | | |

*Note*: Simple Slopes for the Spirituality × Concept Concreteness × Concept Type interaction (Study 2): Slope for spirituality predicting sensorimotor associations for concepts with low concreteness (-1SD): spiritual concepts, *β = .*15, 95% CI [.07, .24], *SE* = .05, *t =* 3.41, *p < .*001, and general concepts, *β = .*10, 95% CI [.01, .19], *SE* = .04, *t =* 2.17, p < .024; at the mean: spiritual concepts: *β = .*08, 95% CI [-.01, .17], *SE* = .04, *t =* 1.78, *p = .*075, and general concepts: *β = .*05, 95% CI [-.04, .14], *SE* = .04, *t =* 1.16, *p = .*247; and high concreteness (+1SD): spiritual concepts, *β = .*01, 95% CI [-.09, .10], *SE* = .05, *t = .*12, *p = .*907; general concepts: *β = .*01, 95% CI [-.08, .09], *SE* = .05, *t =* 0.14, *p = .*889.

**Supplemental Table 8**

*Predictors of Motor Associations Controlling for Modalities (Reference Category: Head No Mouth) (Study 2).*

|  | **Motor Associations** | | |
| --- | --- | --- | --- |
| *Predictors* | *β* | 95*% CI* | *p* |
| Spirituality | 0.12 | 0.01 – 0.22 | **.035*** |
| Concept Type (General: 0, Spiritual: 1) | 0.14 | 0.07 – 0.21 | **.001*** |
| Concept Concreteness | 0.06 | -0.02 – 0.14 | .172 |
| Modality: Hand/Arm | -0.30 | -0.33 – -0.27 | **.001*** |
| Modality: Foot/Leg | -0.65 | -0.68 – -0.62 | **.001*** |
| Modality: Torso | -0.57 | -0.60 – -0.54 | **.001*** |
| Modality: Mouth/Throat | -0.51 | -0.54 – -0.48 | **.001*** |
| Religiosity | 0.02 | -0.09 – 0.12 | .751 |
| Conservatism | -0.08 | -0.16 – 0.01 | .073 |
| Age | -0.02 | -0.10 – 0.06 | .628 |
| Gender (1: Women) | 0.27 | 0.07 – 0.47 | **.008*** |
| Spirituality × Concept Concreteness | -0.00 | -0.01 – 0.01 | .393 |
| Spirituality × Concept Type | -0.04 | -0.06 – -0.03 | **.001*** |
| Concept Concreteness × Concept Type | 0.09 | 0.01 – 0.17 | **.026*** |
| Spirituality × Concept Concreteness  × Concept Type | 0.01 | -0.00 – 0.02 | 0.093 |
| **Random Effects** | | | |
| σ^2^ | 0.64 | | |
| τ_00_ _ps_ | 0.16 | | |
| τ_00_ _item_ | 0.07 | | |
| ICC | 0.27 | | |
| N _ps_ | 96 | | |
| N _item_ | 60 | | |
| Observations | 28800 | | |
| Marginal R^2^ / Conditional R^2^ | 0.108 / 0.345 | | |

**Supplemental Table 9**

*Predictors of Sensory Associations Controlling for Modalities (Reference Category: Interoception) (Study 3).*

|  | **Sensory Associations** | | |
| --- | --- | --- | --- |
| *Predictors* | *β* | 95*% CI* | *p* |
| Spirituality | 0.21 | 0.12 – 0.30 | **.001*** |
| Concept Concreteness | 0.06 | -0.06 – 0.18 | .331 |
| Modality: Touch | -0.07 | -0.10 – -0.03 | **.001*** |
| Modality: Hearing | 0.36 | 0.33 – 0.39 | **.001*** |
| Modality: Seeing | 0.16 | 0.13 – 0.19 | **.001*** |
| Modality: Smelling | -0.44 | -0.47 – -0.40 | **.001*** |
| Modality: Tasting | -0.49 | -0.52 – -0.46 | **.001*** |
| Religiosity | -0.03 | -0.13 – 0.06 | .499 |
| Conservatism | 0.02 | -0.05 – 0.10 | .534 |
| Age | -0.06 | -0.13 – 0.02 | .148 |
| Gender (1: Women) | 0.02 | -0.19 – 0.23 | .850 |
| Spirituality x Concept Concreteness | -0.01 | -0.02 – 0.00 | .073 |
| **Random Effects** | | | |
| σ^2^ | 0.57 | | |
| τ_00_ _ps_ | 0.19 | | |
| τ_00_ _item_ | 0.12 | | |
| ICC | 0.35 | | |
| N _ps_ | 137 | | |
| N _item_ | 30 | | |
| Observations | 24660 | | |
| Marginal R^2^ / Conditional R^2^ | 0.134 / 0.438 | | |

**Supplemental Table 10**

*Predictors of Motor Associations Controlling for Modalities (Reference Category: Head No Mouth) (Study 3).*

|  | **Motor Associations** | | |
| --- | --- | --- | --- |
| *Predictors* | *β* | 95*% CI* | *p* |
| Spirituality | 0.25 | 0.16 – 0.34 | **.001*** |
| Concept Concreteness | 0.07 | -0.05 – 0.18 | .268 |
| Modality: Hand/Arm | -0.07 | -0.10 – -0.04 | **.001*** |
| Modality: Foot/Leg | 0.10 | 0.06 – 0.13 | **.001*** |
| Modality: Torso | 0.52 | 0.49 – 0.56 | **.001*** |
| Modality: Mouth/Throat | 0.05 | 0.02 – 0.09 | **.002*** |
| Religiosity | -0.12 | -0.21 – -0.02 | **.016** |
| Conservatism | 0.02 | -0.06 – 0.10 | .624 |
| Age | -0.06 | -0.14 – 0.01 | .101 |
| Gender (1: Women) | -0.10 | -0.32 – 0.12 | .367 |
| Spirituality × Concept Concreteness | -0.02 | -0.04 – -0.01 | **.001*** |
| **Random Effects** | | | |
| σ^2^ | 0.59 | | |
| τ_00_ _ps_ | 0.20 | | |
| τ_00_ _item_ | 0.10 | | |
| ICC | 0.34 | | |
| N _ps_ | 137 | | |
| N _item_ | 30 | | |
| Observations | 20550 | | |
| Marginal R^2^ / Conditional R^2^ | 0.094 / 0.400 | | |

**Supplemental Table 11**

*Predictors of Sensory Associations Controlling for Modalities (Reference Category: Interoception) (Study 4).*

|  | **Sensory Associations** | | |
| --- | --- | --- | --- |
| *Predictors* | *β* | 95*% CI* | *p* |
| Spirituality | 0.24 | 0.18 – 0.31 | **.001*** |
| Concept Concreteness | 0.22 | -0.01 – 0.45 | .066 |
| modality f1 | -0.05 | -0.09 – -0.01 | **.024*** |
| modality f2 | -0.06 | -0.11 – -0.02 | **.005*** |
| modality f3 | 0.19 | 0.15 – 0.23 | **.001*** |
| modality f4 | -0.34 | -0.39 – -0.30 | **.001*** |
| modality f5 | -0.65 | -0.69 – -0.61 | **.001*** |
| Religiosity | -0.06 | -0.12 – 0.01 | .091 |
| Conservatism | -0.01 | -0.06 – 0.05 | .761 |
| Age | -0.03 | -0.08 – 0.03 | .339 |
| Gender (1: Women) | -0.00 | -0.05 – 0.05 | .935 |
| Spirituality × Concept Concreteness | -0.02 | -0.03 – -0.00 | **.018*** |
| **Random Effects** | | | |
| σ^2^ | 0.53 | | |
| τ_00_ _ps_ | 0.23 | | |
| τ_00_ _item_ | 0.08 | | |
| ICC | 0.37 | | |
| N _ps_ | 351 | | |
| N _item_ | 6 | | |
| Observations | 12636 | | |
| Marginal R^2^ / Conditional R^2^ | 0.165 / 0.477 | | |

**Supplemental Table 12**

*Predictors of Motor Associations Controlling for Modalities (Reference Category: Head No Mouth) (Study 4).*

|  | **Motor Associations** | | |
| --- | --- | --- | --- |
| *Predictors* | *Estimates* | *CI* | *p* |
| Spirituality | 0.22 | 0.16 – 0.29 | **.001*** |
| Concept Concreteness | 0.29 | 0.06 – 0.53 | **.014*** |
| Modality: Hand/Arm | 0.10 | 0.06 – 0.15 | **.001*** |
| Modality: Foot/Leg | 0.32 | 0.28 – 0.37 | **.001*** |
| Modality: Torso | 0.56 | 0.51 – 0.61 | **.001*** |
| Modality: Mouth/Throat | 0.08 | 0.03 – 0.12 | **.002*** |
| Religiosity | -0.02 | -0.09 – 0.04 | .490 |
| Conservatism | 0.01 | -0.04 – 0.06 | .673 |
| Age | -0.04 | -0.09 – 0.01 | .142 |
| Gender (1: Women) | -0.03 | -0.08 – 0.02 | .288 |
| Spirituality × Concept Concreteness | -0.03 | -0.04 – -0.01 | **.001*** |
| **Random Effects** | | | |
| σ^2^ | 0.52 | | |
| τ_00_ _ps_ | 0.22 | | |
| τ_00_ _item_ | 0.09 | | |
| ICC | 0.37 | | |
| N _ps_ | 351 | | |
| N _item_ | 6 | | |
| Observations | 9005 | | |
| Marginal R^2^ / Conditional R^2^ | 0.173 / 0.480 | | |
